# Behind the Mask: Parkinson's Disease and Depression

**DOI:** 10.7759/cureus.52663

**Published:** 2024-01-21

**Authors:** Sneha Balasubramanian, Khawar Tariq Mehmood, Shahad Al-Baldawi, Gabriel Zúñiga Salazar, Diego Zúñiga

**Affiliations:** 1 Internal Medicine, Madras Medical College, Chennai, IND; 2 Internal Medicine, Aster Hospital Br of Aster DM Healthcare FZC, Dubai, ARE; 3 Rheumatology, Al-Yarmouk Teaching Hospital, Baghdad, IRQ; 4 Medicine, Universidad Católica de Santiago de Guayaquil, Guayaquil, ECU

**Keywords:** non-motor symptoms, neurodegeneration, anti-depressants, electro-convulsive therapy, cognitive behavioural therapy, pimavanserin, neuropathology, treatment of depression in parkinson's disease, depression, parkinson' s disease

## Abstract

Parkinson's disease (PD) is a common, prevalent neurodegenerative disease. It is mainly characterized by motor symptoms such as rigidity, tremors, and bradykinesia, but it can also manifest with non-motor symptoms, of which depression is the most frequent. The latter can impair the quality of life, yet it gets overlooked and goes untreated because of the significant overlap in their clinical features, hence making the diagnosis difficult. Furthermore, there is limited data on the availability of appropriate criteria for making the diagnosis of depression in PD patients, as it can occur with varying expressions throughout the course of PD or it can also precede it. This review article has included a brief discussion on the diagnosis of depression in PD patients and their overlapped clinical manifestations. Understanding the mechanisms underlying the disease processes of PD and depression and the pathways interconnecting them gives better knowledge on devising treatment options for the patients. Only studies from Pubmed were included and all other databases were excluded. Studies from the last 50 years were included. Suitable references included in these studies were also extracted. Thus, depression in PD and PD in depression, along with their pharmacological and non-pharmacological treatment options, have been discussed.

## Introduction and background

Parkinson’s disease (PD) is a common chronic progressive degenerative neurologic disorder. After Alzheimer’s disease, it is the second most common neurodegenerative condition worldwide. PD affects 1% of the population over 60 years old, and its prevalence keeps increasing with age. With the aging population, by 2050, it is predicted that the prevalence of PD will double. Genetic factors, environmental pollutants, and aging are possible causes of the disease; however, the etiology and pathogenesis of PD remain unclear [1-4]. While the precise cause of PD is unknown, it is thought that the build-up of Lewi bodies, which are mainly made of synuclein protein, and mitochondrial dysfunction and defective proteolysis in areas such as the substantia nigra, are potential contributors to neuronal cell deaths and the consequent decrease in striatal dopamine levels seen in PD brains [5]. Although the diagnosis of PD primarily depends on motor symptoms such as tremors, rigidity, and bradykinesia, it can also present as non-motor symptoms.

Neuropsychiatric, autonomic, and sensory symptoms are different types of non-motor symptoms. The various neuropsychiatric symptoms that the patients experience include depression, anxiety, hallucinations, sleep disturbances, and cognitive impairment, of which depression is the most common. Reduced facial expressions, trouble sleeping, weariness, psychomotor slowness, and decreased appetite are symptoms that both depression and PD share. In individuals who have PD, clinically severe depression is reported to occur 40-50% of the time [6-8]. According to a number of neurobiological studies, depression in PD may result from dopamine and adrenaline innervation declines in the limbic system and striation, in addition to serotoninergic abnormalities [9]. The standard criteria from the Diagnostic and Statistical Manual of Mental Disorders (DSM) are used to make its diagnosis [10]. The identical symptoms of PD and depression could lead to a depression underdiagnosis and can thereby go untreated in PD patients. It is also estimated that only around 20% of individuals who are diagnosed with depression in PD patients receive treatment [11]. Pharmaceutical and non-pharmacological treatment options are currently used in the clinical management of depressive disorders in PD patients [12]. Because pharmacological treatment has its limitations, non-pharmacological remedies are slowly gaining popularity. A number of non-pharmacological therapies are now used to treat depressive symptoms in people with PD. These therapies can be roughly divided into physical exercises, traditional Chinese exercises, supportive therapies (such as yoga, acupuncture, dance, and music therapy), and others, such as cognitive behavioral therapy (CBT), electroconvulsive therapy (ECT), cognitive training, psychotherapy, bright light therapy (BLT), deep brain stimulation (DBS), and trans-cranial magnetic or direct current stimulation, a few to mention [13].

If left untreated, depression has effects that go far beyond the symptoms of the mood. This could be severe functional impairment, quicker physical and cognitive decline, higher mortality, lower quality of life, and greater stress for caregiving. All this makes it pivotal to remind patients, their families, and other staff that depression in PD is treatable and remission is achievable [14]. This review article, therefore, intends to emphasize the interplay of pathophysiological pathways of depression in PD patients, highlight the diagnostic methods, and evaluate the multifarious treatment options for them.

## Review

Neural Mechanisms Interconnecting PD and Depression

**Figure 1:**
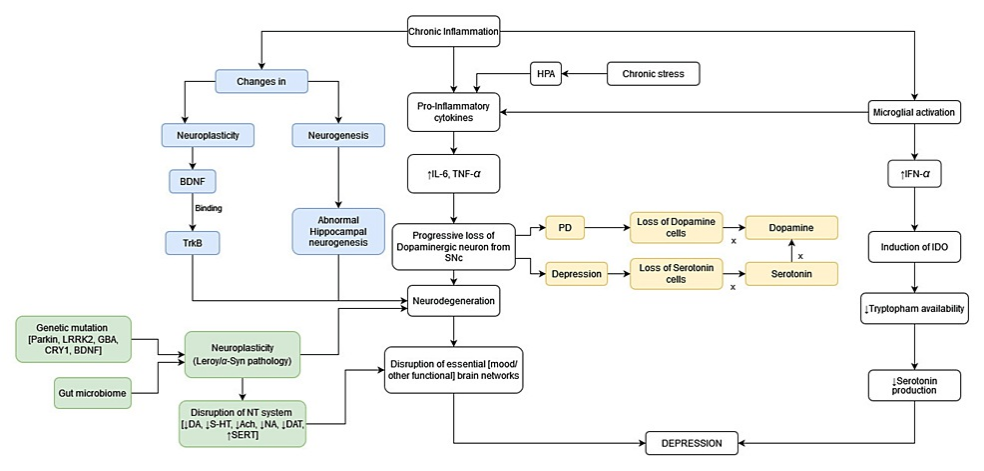
Summary of the pathogenesis HPA – Hypothalamus-pituitary-axis, BDNF – Brain-derived neurotrophic factor, IL-6 – Interleukin-6, TNF-alpha – Tumor necrosis factor-alpha, IFN-gamma – Interferon-gamma, IDO – indoleamine 2,3 dioxygenase, PD – Parkinson’s disease, LRRK 2 – Leucine-rich repeat kinase 2, GBA – Glucocerebrosidase, CRY1 – Cryptochrome circadian regulator 1, DA – Dopamine, 5-HT – Serotonin, Ach – Acetylcholine, NA – Nor-adrenaline, DAT – Dopamine transporter, SERT – Serotonin transporter Image credits: Khawar Tariq Mehmood

The pathophysiological mechanisms concerning PD and depression are complex and multifaceted, involving various neurobiological processes such as the neurodegenerative process of PD, deterioration in the levels of neurotransmitters (such as dopamine (DA), serotonin (5-HT), noradrenaline (NA), and acetylcholine (Ach)), neuroinflammation, and neuroplasticity (Figure 1) [15]. To address the broad spectrum of PD depression and provide customized treatment options to treat depression in all stages, it is of fundamental importance to understand the pathophysiological mechanisms and the role of dopaminergic and non-dopaminergic pathology in people with PD [16].

The characteristic neuropathological lesions of PD are the degeneration of dopaminergic neurons and intraneuronal Lewy bodies. However, the loss of distinct Nor-adrenergic and serotonergic neurons in PD emphasized that the neurological disease extends beyond the midbrain. Collectively, these neuronal networks are responsible for the control of the mood and reward systems and concert the mood variations in not only PD patients but also the general public [9]. Based on a prevalent concept, it is stated that mesocortical and mesolimbic dopaminergic neuronal degeneration leads to orbitofrontal dysfunction, which, in turn, causes disruption in the serotonergic neurons in the dorsal raphe and further progresses to the dysfunction of the depression-related orbitofrontal-basal ganglia-thalamic networks [17]. Neurotransmitter changes occur before dopaminergic neurodegeneration and have a significant impact on the development of nonmotor symptoms. Depression-like symptoms in PD patients are seen due to reduced dopamine transporter (DAT) availability as per the positron emission tomography (PET) scans of the striatal and limbic areas. Additionally, it was found that the levels of dopamine in cerebrospinal fluids (CSF) were considerably decreased in PD patients with depression compared to those of non-depressed patients with PD. Moreover, serotonin transporter (SERT) levels worsened the depression symptoms, and studies revealed that raphe nuclei and limbic structures showed increased SERT binding in PD patients with depression than those without depressive symptoms. The reduction in the levels of 5-HT and its metabolite 5-hydroxyindoleacetic acid (5-HIAA) also corresponds to the severity of depressive symptoms in PD patients [18-21]. The noradrenergic impairment connected to PD depression and anxiety was identified in the thalamus and bilateral locus coeruleus, as well as the right amygdala, using PET imaging. An outcome suggests that cortical cholinergic depletion was relevant to the depression score seen in mid-stage demented and non-demented male PD patients [18,22]. Furthermore, a reduced level of Ach receptor binding in the cortex of depressed patients with PD was observed [23]. Dopamine is inhibited by both the serotonergic and cholinergic systems; in the initial stages, the decrease in the activity of the serotonergic and cholinergic systems can temporarily make up for the dopaminergic deficits. However, to a lesser extent, cholinergic and noradrenergic neurons gradually deteriorate. These mechanisms can contribute to the development of depression and tell us that depression frequently precedes motor symptoms. Concurrently, the dysfunction of the mesocorticolimbic dopaminergic system correlates to the severity of depression in PD patients. Therefore, there is enough evidence to suggest that PD depression is related to pathological processes beginning in the prodromal state and that the evolution of dopaminergic and non-dopaminergic pathology over the stages of PD elucidates the varying expression and course of depression over time. This is also further substantiated by the Braak staging (Table 1), which suggests that nonmotor symptoms, such as anosmia, depression, anxiety, and cognitive changes, appear before the onset of motor symptoms [24-27].

**Table 1:** Braak Staging

Stages	Pathology	Outcomes	Symptoms
Stage one	Dorsal motor nucleus of the vagal nerve, Anterior olfactory structures	Olfactory loss, autonomic dysfunction	Non-motor
Stage two	Lower raphe nuclei, locus coeruleus	Affective impairment, anxiety, sleep disturbance, depression	
Stage three	Substantia nigra, Amygdala	Motor symptoms – clinical diagnosis	Motor
Stage four	Temporal mesocortex	Worsening motor symptoms, emotional disturbances	
Stages five and six	Temporal neocortex sensory association and pre-motor areas	Worsening motor symptoms cognitive changes	

Inflammation is one of the mechanisms of neurodegeneration and a converging point for major depressive disorder (MDD) and PD. It was identified by the presence of activated microglial cells in the substantia nigra pars compacta and increased levels of proinflammatory cytokines in the brain and blood in PD patients [28,29]. Immune dysregulation leading to increased production of proinflammatory cytokines such as interleukin-6 (IL-6) and tumor necrosis factor-alpha (TNF-alpha) in the blood and brain, which is released due to microglial and activated astrocytes, causes the worsening of the degeneration of dopaminergic neurons and subsequent alteration in brain function [28-30]. Decreased levels of 5-HT in the brain lead to depression. This is mediated by an extensive pathway whereby interferon-gamma (IFN-gamma) induces the enzyme indoleamine 2,3-dioxygenase (IDO), which leads to a significant tryptophan availability reduction and finally results in reduced 5-HT levels [31]. The affected region of the brain demonstrates the buildup of immune cells from the periphery. In PD, misfolded variants of the alpha-synuclein protein activate microglia and astrocytes, which, in turn, generate cytokines that react with multiple physiological processes to cause depressive symptoms. Added to this are the stress-induced hypothalamus-pituitary axis, monoamine transport, and hippocampal neurogenesis, which activate this process. The lymphatic clearance process establishes that astrocytes are another important link connecting PD and depression. Studies show that MDD leading to astrocyte degeneration or structural atrophy in the essential region of the brain indicates that alpha-synuclein accumulation in certain parts of the brain is due to the deficient clearance of these protein aggregates, which are pathogenic. These processes underlie the fact that MDD and PD share a common pathophysiology, which is neuroinflammation [32]. In addition to this, inflammation serves as an integral part of microbiome alteration, which may also be a contributing factor to depression in PD [33]. The epigenetic alterations in leucocytes and neurons in PD patients and the severity of depressive symptoms are in accord with the reduced levels of butyrate contributed by fecal bacteria [34].

Theories of neurogenesis and neuroplasticity have emerged to address the limitations of the monoamine hypothesis and theories focusing only on neurotransmitters. A significant alternate hypothesis states that, when comparing healthy subjects to depressed patients, there was a reduction in hippocampal volume, in accordance with the meta-analysis of magnetic resonance imaging (MRI) studies [35]. The neuroplasticity theory concentrates on shortened dendrites, reduced spine numbers, density, and such morphological alterations, while the neurogenesis hypothesis relies on decreased hippocampal neurogenesis. Together, they highlight the fact that there is a decrease in hippocampal volume in depressed patients [26]. A widely recognized growth factor, brain-derived neurotrophic factor (BDNF), works as a crucial anti-depressant. It binds with tropomyosin receptor kinase B (TrkB) with high affinity and produces BDNF-TrkB signaling, which governs neurotransmission, augments synaptic efficiency, and overlooks the processes of neuronal differentiation, maintenance, regeneration, and survival. Guilloux et al. stated in their study that postmortem brains of depressed patients exhibit reduced BDNF and Trk B expression. Jiang et al., in their study on PD patients, stated that there are decreased levels of serum BDNF in PD patients. The above-mentioned studies, therefore, establish the link between BDNF and neuroplasticity in the pathogenesis of depression in PD patients [36-38].

In patients with genetic PD, depression is frequently found. Of particular importance is the identified risk factor for early-onset PD, the Parkin mutation. Relatives without diagnosed PD, but with heterogenous mutation, have an increased probability of depression than relatives without mutation in Parkin [39]. The mutation carriers had a longer disease duration, along with an early onset and an increased index of depression, all suggesting that the Parkin mutation correlates well with the symptoms of depression without affecting the executive functions of PD. The carriers of leucine-rich repeat kinase 2 (LRRK 2) had associations with depression and hallucinations, which points toward limbic system involvement in these patients [40,41]. LRRK 2 patients with manifest PD had depression of close to 30%, and it may predate motor symptoms such as those of idiopathic PD patients [42]. Additionally, at high risk of PD are heterozygous glucocerebrosidase (GBA) mutation carriers, whose severity of depression increased over two years. It is also found with severe non-motor symptoms such as cognitive and olfactory impairment [43]. Cryptochrome circadian regulator 1 (CRY 1) and BDNF mutations are also frequently found with depressive symptoms in PD patients [44]. Hence, there is booming data regarding the pathways and mechanisms that PD and depression share.

Overlap in the Clinical Manifestations of PD and Depression With the Enigma of Diagnosis

**Figure 2:**
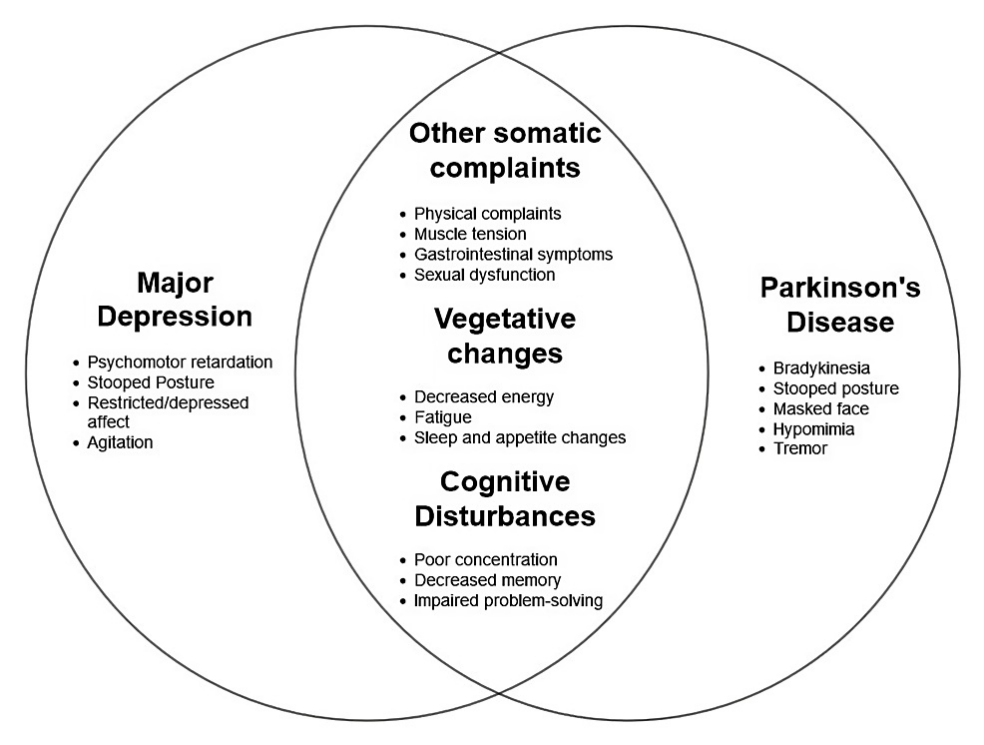
Overlapping Clinical Features Image credits: Khawar Tariq Mehmood

The challenges put forth by PD and the increased age of the patients lead to the masking of the depressive symptoms, as expected, and the overlap of the symptoms between MDD and PD because both have features of fatigue, loss of energy, reduced appetite, insomnia, concentration difficulties, retarded psychomotor activities, a decline in intellectual functions, hypomimia, and an inability to experience pleasure (anhedonia). The patient can assume a depressed affect as the range of facial expressions can be limited by akinesia. Similarly, psychomotor retardation in depression can feature like hypophonia and bradykinesia. Due to their disability, chronicity, or social embarrassment regarding their situation, patients may have withdrawn from their usual day-to-day activities (Figure 2). This feature is like a coin with two sides, as it can either mask the underlying depression or lead to undertreatment of PD due to overdiagnosis of depression by clinicians. Major depression, minor depression, and dysthymia are the main subtypes of depression diagnosed in PD patients. It is observed to be akin to depressive subtypes in the non-PD population. PD-specific features such as the progression of the disease, which is not limited by treatment or the effect of medication on mood and motor features, along with increased psychiatric influences, make the assessment of depressive symptoms in PD patients complicated. Especially during the off-state of PD, the depressive symptoms are more severe and frequently occurring [12,45,46].

As mentioned above, it is difficult to assess depression in PD patients because of the overlapping features; henceforth, it is crucial to inquire directly about the mood phenomena in PD patients, or else the diagnosis can be overlooked, leading to deferment of treatment [47]. A multicultural community study evaluated that the most practical way of diagnosing PD is through the assessment of clinical features after excluding all other causes of PD. Utilizing at least two features - rigidity, bradykinesia, or resting tremors - allows considerable accuracy of diagnosis, and prevalence estimation was established by the study [48]. Somatic features that were found to be overlapping belonged to depressive disorders and not to PD alone. Hence, the National Institute of Health recommends an inclusive diagnostic approach. Several studies suggest that, for establishing the diagnostic criteria for major depression, minor depression, and dysthymia in PD, the fourth edition of the American Psychiatric Association's Diagnostic and Statistical Manual (DSM-IV) can be used [49]. The features of depressive disorders go beyond the nine DSM-IV criteria; only four are affective, and the remaining is somatic. Because the somatic symptoms cause an overlap between PD and depressive symptoms, clinicians must concentrate on emotional symptoms when compared to the neurovegetative symptoms to arrive at the diagnosis of depressive disorders. Alternatively, major depression without neurological disease has a diminished ability to perceive pleasure (anhedonia), declined interest levels, or pervasive and persistent low moods [10]. Screening and identification can be helped by the clinician and self-reporting scale, but they are not to be used in lieu of a diagnostic clinical examination. In patients with PD, many self-reported depressive screening tools have beneficial psychometric components and have proved to be valuable predictors of major and non-major depression; however, a scale is chosen largely because of its simplicity of use. Along the same lines, because it is available freely (not copyrighted), has psychometric features, and is relatively easy to use, the Geriatric Depression Scale (GDS) was recommended among the nine other scales to detect PD. The GDS-30 questionnaire has a simple format of 30 items, which could be easily filled out by the patients themselves. The most discriminative value was found to be the cut-off score of 9/10. However, GDS-15 may be even more optimal because it relies less on the physical symptoms, which overlap with PD symptoms and are commonly featured in other scales used to find depressive symptoms in the general population. It was also established to have good positive predictive value and high sensitivity when used for diagnostically validated depressive symptoms in PD [50-52]. The depression scales can be used to monitor the effectiveness of treatment at regular clinical follow-ups once a depressive disturbance has been detected. A thorough physical examination and laboratory screening should be offered to patients with PD and depression so that systemic causes of depression such as thyroid or liver dysfunction or vitamin B12 or testosterone deficiency can be ruled out. Moreover, it is worth noting that the treatment regimen for PD can cause mood disturbances, so adjustments can be made to reduce fluctuations that can be related to mood lability [45].

Treatment

PD and depression, with their complex and overlapping pathophysiology and clinical features, pose a major problem in getting themselves identified and treated. Because depression affects daily activities, causes a decline in cognitive levels, and negatively impacts the quality of life, it is pertinent to treat it as much as PD itself. With the inherent stressors in PD, it may be unclear whether the patient has a mood disorder or not. This needs watchful waiting and strategies of problem-solving as a reasonable first step, with a follow-up within two to three weeks and not later than that. When the symptoms of depressive disorders in PD become persistent and lead to impairment or distress, treatment is warranted. Because of the heterogeneity of depression in PD, the treatment offered should contain multimodal interventions that are customized to each individual's needs. The various approaches include education about PD and the mood disturbances associated with it, emotional support, appropriate medication, and help with acquiring coping strategies for mood symptoms. Early initiation of physical, occupational, and speech therapies, home care services, and the involvement of social care workers to educate about PD to patients and their caregivers could reduce the stressor burden and help them combat depression or anxious moods. Along with these enrollments in peer support groups, exercise, good sleep hygiene, and healthy emotional activities should be motivated. Supportive psychotherapy, which encourages patients to engage in personal and social activities, is recommended for the treatment of mild depression. Pharmacological treatment is validated for cases of severe depression [45,53,54].

Pharmacological Interventions

Tricyclic anti-depressants (TCAs), selective serotonin receptor inhibitors (SSRIs), serotonin and norepinephrine reuptake inhibitors (SNRIs), and monoamine oxidase B (MAO B) inhibitors are some of the most commonly investigated groups of drugs for depression in PD patients.

The mechanism of action of TCAs is that they prevent the reuptake of serotonin and noradrenaline and cause long-term elevated receptor sensitivity. They have been traditionally used to treat depression symptoms in the general population, but only a small number of studies have been done on their actions in PD patients with depression. Although it has been used as a first-line agent for depression for many years successfully, it has now been surpassed by SSRIs/SNRIs because of its low therapeutic index and its unfavorable profile of side effects due to its anti-cholinergic and anti-adrenergic properties (unacceptable in the vulnerable PD population), which lead to delirium, dysautonomia, and orthostasis. However, clinicians must take note that it is still considered a useful alternative to resistant depressive symptoms in PD [55-59].

Paroxetine, sertraline, citalopram, escitalopram, and fluoxetine are some of the SSRIs, while venlafaxine and duloxetine are some of the newer SNRIs that are used to treat depression in non-PD patients as first-line agents. Both significantly improve depression symptoms. This is supported by the study done by Richard et al. in 2012. The study comprised 115 subjects with depression in PD patients in a randomized, placebo-controlled, double-blind study lasting 12 weeks with paroxetine 40 mg/day, venlafaxine extended-release (XR) 225 mg/day, and placebo using the DSM-IV and Hamilton Rating Scale for Depression (HAM-D) as diagnostic criteria. The study concluded that both paroxetine and venlafaxine significantly improve depression in PD patients compared to placebo. It was also found that both drugs were well-tolerated and safe and did not heighten the motor symptoms of PD [60]. However, some evidence states that TCA (nortriptyline) was superior to placebo and not SSRI (paroxetine) when nortriptyline, paroxetine, and placebo were involved in the study. The study by Menza et al. in 2009 was a randomized controlled trial (RCT) of paroxetine controlled-release (CR), nortriptyline, and placebo for eight weeks in 52 patients with depression and PD using the HAMD as a diagnostic criterion. The interpretation of the study was that nortriptyline was more efficacious and paroxetine was not when compared to placebo [56]. Roughly similar, another study by Devos et al., consisting of TCA (desipramine) and SSRI (citalopram), in a randomized placebo-controlled study of 48 patients with depression in PD with the Montogomery-Asberg Depression Rating Scale (MADRS) as a diagnostic criterion, concluded that after 14 days of treatment initiation, desipramine was efficacious than citalopram and placebo, but after 30 days, both desipramine and citalopram proved to be more efficacious than placebo [57].

A drug primarily used for motor symptom management but also proved efficient in the treatment of depression in PD patients is dopamine-agonist pramipexole. It can be considered an alternate treatment for depression in PD patients. Its efficiency has been compared to SSRIs. Barone et al. conducted a randomized multicenter parallel-group study for 14 weeks with pramipexole and sertraline with HAM-D as diagnostic criteria and demonstrated that both showed significant improvement in depression symptoms [61,62].

Monoamine oxidase (MAO) inhibitors are of two types: type A and type B. Type A inhibitors affect the NA and 5-HT pathways, whereas type B blocks dopamine catabolism. Selegiline and rasagiline are some MAO-B inhibitors. There are several studies done on them for their clinical usefulness in depression. Smith et al. conducted a double-blind placebo-controlled trial of rasagiline in PD patients and proved a favorable outcome to rasagiline when assessed against placebo in both depression and cognitive scores. Whereas in the same year 2015, Barone et al., in a randomized double-blind placebo-controlled trial using the Beck Depression Inventory (BDI), the Unified Parkinson's Disease Rating Scale (UPDRS), and the Parkinson's Disease Quality-of-Life Questionnaire-39 (PDQ-39), concluded that rasagiline had no significant effect compared to placebo on depression symptoms [63,64].

Newer and Other Pharmacological Interventions

Trazadone, a 5-HT-2a/c antagonist, significantly improved depression; not only that, it enhanced motor functions in depressed patients. This is supported by the study of Werneck et al. in 2009, using HAMD and UPDRS as diagnostic criteria, which established that trazodone helps in depression in PD because of the 5-HT (hydroxytryptophan) 2c receptors antidopaminergic properties [65].

Pimavanserin, another atypical antipsychotic with an antagonist and inverse agonist at the 5-HT 2a receptor, is also found to have positive effects on depression in PD, as stated by Dekraske et al. in 2020 in a single-arm open-label study with HAMD-17 as its diagnostic criterion. It is also suggested that it can be used as an adjunctive to SSRI/SNRI or even as monotherapy [66].

Several studies have shown that non-motor symptoms, such as apathy and depression associated with PD, have altered serotonergic neurotransmission. Based on this, Meloni et al. did a single-center randomized double-blind control crossover trial in 2020 for over four weeks with 50 mg of 5-HTP and found that, with the BDI and Hamilton Depression Rating Scale (HDRS) as diagnostic criteria, 5-HTP was better when compared to placebo [67].

Dietary supplements with antidepressant properties: A double-blind randomized placebo control study in 2008 on fish oil (omega-3 fatty acids) using MADRS, BDI, and the Clinical Global Impressions Scale (CGI) as diagnostic criteria proved to improve depressive symptoms in PD patients. However, limited studies are present on this dietary supplement [68].

Non-pharmacological Interventions

On experimentation, it was found that transcranial magnetic stimulation (TMS) reduced depressive symptoms temporarily. Specific brain regions, when subjected to magnetic and electrical energy, caused only functional changes and no structural derangements. Patients with PD experienced transient changes in both mood and motor functions. A recent study done in 2022 by Chen et al. compared routine treatment, escitalopram, pramipexole, and TMS in an open-label randomized control trial with HAMD as diagnostic criteria and found that escitalopram, pramipexole, and high-frequency TMS had better efficacy in patients with depression in PD when compared to routine anti-PD drugs (levodopa and benserazide) [69,70].

The effects of electroconvulsive therapy (ECT) have an impact on both the dopaminergic and serotonergic pathways. In animals, it was found that ECT increased dopamine transmission, which was probably attributed to the increased activity of endogenous MAO inhibitors. Its serotonergic effects are due to the upregulation of postsynaptic 5-HT2 receptors and the downregulation of presynaptic 5-HT1a receptors. ECT has demonstrated effective antidepressant properties but requires cautious use in patients with PD. It is of particular importance to patients with suicidal ideation or those who are awaiting a response to antidepressant medications [71,72]. A systematic review and meta-analysis study of around 14 previous studies on the efficacy of ECT on PD symptoms in 2021 concluded that ECT improved depression and psychiatric symptoms, as well as the motor components in PD patients [73].

For mild-to-moderate depression in PD, in addition to medication alteration, CBT can be used as a first-line or supplementary treatment. In a study, traditional clinical treatment was compared to patients subjected to CBT, which consisted of cognitive restructuring, anxiety coping techniques with worry control, behavioral activation, sleep hygiene protocols, and caregiver support for 14 weeks. It summarized that symptom severity improved by 56% for PD depression patients (of whom some were consuming antidepressants), while it was 8% for the control group [74]. Table 2 presents a comparison of a few important pharmacological and non-pharmacological treatment studies in the past 20 years that are included in this study.

**Table 2:** Comparison of a Few Important Pharmacological and Non-pharmacological Treatment Studies in the Past 20 Years Included in This Paper PD - Parkinson’s disease, RCT - Randomized clinical trial, TMS - Transcranial magnetic stimulation, HAM-D - Hamilton depression rating scale, BDI - Beck depression inventory, UPDRS - Unified Parkinson’s disease rating scale, PDQ - Parkinson’s disease quality of life questionnaire, DSM - Diagnostic and Statistical Manual of Mental Disorders, XR - Extended release, CBT - Cognitive behavioral therapy, CR - Controlled release, MADRS - Montgomery-Asberg Depression Rating Scale, CGI - Clinical Global Impressions Scale

Reference	Design	Treatment	Trial duration	Diagnostic criteria	Conclusion
Menza et al. (2009) [56]	RCT	Nortriptyline Paroxetine- CR Placebo		HAM-D	Comparatively, nortriptyline was better than paroxetine in the treatment of depression in PD patients
Richard et al. (2012) [60]	Randomized double-blind placebo-controlled	Paroxetine-40 mg/day Venlafaxine XR-225mg/day	12 weeks	DSM-IV HAM-D	Paroxetine and venlafaxine were safe, well-tolerated, and improved depression symptoms in PD patients
Barone et al. (2006) [62]	Randomized multicenter parallel group	Pramipexole Sertraline	14 weeks	HAM-D	Both showed improvement in depressive symptoms. Pramipexole can be an alternative for depression treatment in PD patients
Barone et al. (2015) [64]	Randomized double-blind placebo-controlled	Rasagiline 1 mg/day	12 weeks	BDI UPDRS PDQ-39	Rasagiline had no significant effects compared to placebo on depressive symptoms in PD patients
Werneck et al. (2009) [65]		Trazodone		HAM-D UPDRS	It improves depression symptoms significantly in PD patients
Dekarske et al. (2020) [66]	Single-arm open-label	Pimavanserin	8 weeks	HAMD-17	Showed improvement of depressive symptoms in PD patients and was also well tolerated
Da Silva et al. (2008) [68]	Randomized double-blind placebo-controlled	Fish oil (omega-3 fatty acid)	3 months	MADRS CGI BDI	It showed an improvement in depressive symptoms in PD patients
Chen et al. (2022) [70]	Open-label RCT	Routine treatment Escitalopram Pramipexole TMS	4 weeks	HAM-D	Escitalopram, Pramipexole and High-frequency TMS had better outcomes than routine treatment for depressive symptoms in PD patients
Dobkin et al. (2011) [74]	RCT	CBT	14 weeks	HAMD-17	CBT improved depression symptom severity in PD patients

Some other somatic treatment options, such as DBS and vagal nerve stimulation, are also being experimented with as alternatives to PD depression management [75].

The encouragement of physical activity in PD may not only be beneficial to global and motor problems, but it also has a positive effect on depression, especially with weekly or biweekly yoga and aerobic training exercises. Exercise increases dopaminergic release in the caudate nucleus, improves mesolimbic function, and facilitates an anti-inflammatory response, thereby proving to be beneficial in ameliorating depression symptoms in PD patients. BLT was also found to be useful for depressive symptoms in PD patients (even though it is primarily used for sleep disorders) in a randomized control study in 2019 [76,77].

Limitations

The non-motor symptoms of PD are various in number. This paper focuses only on the non-motor symptoms of depression and its relation to PD. The pathophysiological mechanisms involving depression and PD are complex and large in number; hence, only the prominent ones are discussed. Similarly, the availability of treatment options being diverse, only a handful of important and recently researched methods are included. This study article is unable to decode the paradox of which came first: depression or PD, due to the underlying features of overlapping pathogenesis, clinical features, and few criteria of diagnosis.

## Conclusions

PD is characterized mainly by its cardinal motor features such as bradykinesia, tremors, and rigidity, but it can also present with its non-motor symptoms, making it equally important to identify and provide treatment to improve the patient’s quality of life. One of the frequent non-motor symptoms of PD, depression can occur with variable expressions anytime along the course of PD, or it can also precede it. Both share interconnected mechanisms involving the dopaminergic, serotonergic, and noradrenergic pathways. This, along with the significant overlap of the clinical features, makes it challenging to diagnose depression in PD patients in a clinical setting. Therefore, screening patients with PD for depression and vice versa helps in the early identification and treatment. As discussed, even with the existence of simplified scales to pick out depression in PD patients, certain shortcomings warrant more research in this area to achieve a more accurate diagnosis. Various studies discussed show the efficacy of pharmacological management, along with non-pharmacological management, which can be used in addition or even as first-line agents in certain conditions such as mild depression per the patient's symptoms. Therefore, the clinical significance of this review article lies in not only exposing depression in PD patients but also in choosing the appropriate treatment from the basket of options available. It is not an unimodal, but rather an integrated and customized treatment plan for each individual according to their presentation and stage of disease, with other influencing variables taken into account. As such, the management of depression in PD patients is challenging. Therefore, more future studies should be performed on the processes interlinking PD and depression, structured and targeted diagnostic approaches, and the timely interventions possible for achieving remission.
